# Qualitative assessment of attitudes and knowledge on preterm birth in Malawi and within country framework of care

**DOI:** 10.1186/1471-2393-14-123

**Published:** 2014-04-02

**Authors:** Judy Levison, Debora Nanthuru, Grace Chiudzu, Peter N Kazembe, Henry Phiri, Susan M Ramin, Kjersti M Aagaard

**Affiliations:** 1 Department of Obstetrics and Gynecology, Baylor College of Medicine, 1 Baylor Plaza, 77030 Houston, TX, USA; 2 Baylor Malawi Center of Excellence, Kamuzu Central Hospital, 265 Lilongwe, Malawi; 3 Department of Obstetrics and Gynecology, Kamuzu Central Hospital, 265 Lilongwe, Malawi; 4 Department of Obstetrics and Gynecology, Division of Maternal-Fetal Medicine, Baylor College of Medicine, One Baylor Plaza, 77030 Houston, TX, USA

**Keywords:** Preterm birth, Qualitative research, Global women’s health, Pregnancy, Periodontal disease

## Abstract

**Background:**

The overarching goal of this study was to qualitatively assess baseline knowledge and perceptions regarding preterm birth (PTB) and oral health in an at-risk, low resource setting surrounding Lilongwe, Malawi. The aims were to determine what is understood regarding normal length of gestation and how gestational age is estimated, to identify common language for preterm birth, and to assess what is understood as options for PTB management. As prior qualitative research had largely focused on patient or client-based focused groups, we primarily focused on groups comprised of community health workers (CHWs) and providers.

**Methods:**

A qualitative study using focus-group discussions, incidence narrative, and informant interviews amongst voluntary participants. Six focus groups were comprised of CHWs, patient couples, midwives, and clinical officers (n = 33) at two rural health centers referring to Kamuzu Central Hospital. Semi-structured questions facilitated discussion of PTB and oral health (inclusive of periodontal disease), including definitions, perception, causation, management, and accepted interventions.

**Results:**

Every participant knew of women who had experienced “a baby born too soon”, or preterm birth. All participants recognized both an etiology conceptualization and disease framework for preterm birth, distinguished PTB from miscarriage and macerated stillbirth, and articulated a willingness to engage in studies aimed at prevention or management. Identified gaps included: (1) discordance in the definition of PTB (i.e.*,* 28–34 weeks or less than the 8^th^ month, but with a corresponding fetal weight ranging 500 to 2300 grams); (2) utility and regional availability of antenatal steroids for prevention of preterm infant morbidity and mortality; (3) need for antenatal referral for at-risk women, or with symptoms of preterm birth. There was no evident preference for route of progesterone for the prevention of recurrent PTB.

**Conclusions:**

Qualitative research was useful in (1) identifying gaps in knowledge in urban and rural Malawi, and (2) informing the development of educational materials and implementation of programs or trials ultimately aimed at reducing PTB. As a result of this qualitative work, implementation planning was focused on the gaps in knowledge, dissemination of knowledge (to both patients and providers), and practical solutions to barriers in known efficacious therapies.

## Background

Malawi has the highest preterm birth (PTB) rates in the world, currently estimated at an overall incidence of 18.1% [1-3]. Of the 4 million newborn deaths annually on a global scale, over 1/4 are directly attribu to prematurity with another 1/3 secondary to prematurity-related infections (sepsis, pneumonia, gastrointestinal such as necrotizing entercolitis; 4). 75% of the 4 million deaths occur within the first week of life, with the vast majority occurring in the first 48 hours [4,5]. In under-resourced rural communities, infant mortality related to preterm and low birth rate birth exceeds that in urban communities, and is not solely attributed to limited access to secondary and tertiary care [4].

The World Health Organization has recognized the “survival gap” between infants born in low and high resource settings: 90% of infants less than 28 weeks survive in high resource countries and 10% survive in low resource settings (http://www.who.int/maternal_child_adolescent/documents/born_too_soon/en/). Recognition of the prevalence of preterm birth in sub-Saharan Africa as well as the survival gap has led to recent good intentioned efforts aimed at both primary prevention and secondary interventions [4]. However, success of these efforts may be hampered by accompanying gaps in our knowledge of the perceptions and framework with which preterm birth is understood among at-risk women and families, as well as regional health care providers and community health workers in such low-resource settings. Furthermore, barriers to successful prevention and intervention may be regional and population specific and cannot be inferred from one community to another [2-5]. Ultimately, the successful acceptance and utilization of any given preterm birth prevention or intervention is dependent upon a communities perception of the underlying prevalence, etiology, attributable risk-factors, and in-country framework for care.

Qualitative research seeks to address the “*whys* and *hows*” and thereby complements the “*how much* and *how many*” approach of quantitative research [6]. Specifically, qualitative methods offer potential for understanding disease concepts and perceptions among the spectrum of community members, including those who are socially disadvantaged and advocate limited [5,6]. This occurs by enabling each member to express their reality in a non-judgmental, active-listening setting [6-9]. Kumbani et al. have used qualitative methods to assess women’s perceptions of general perinatal care in Malawi [10]. However, a review of the literature (PubMed, 1996–2013) revealed only one previous qualitative study of women’s perceptions of preterm birth in Malawi [11]. Those authors examined community members’ (women, men, and health care workers including traditional birth attendants) conceptualizations of preterm birth, perceived causes, and potential behavioral associations. However, these authors did not concomitantly assess any current or potential preventative or interventional strategies aimed at reducing the burden of preterm birth and did not develop any health care messages aimed at reducing the identified knowledge gap.

At the start of a funded preterm birth primary prevention initiative in a rural–urban setting in Lilongwe, Malawi (Saving Lives at Birth: A Grand Challenge for Development USAID AID-OAA-G-11-00062), we sought to complete a needs assessment and gap analyses employing validated qualitative field research methodologies, then develop health care messages which address identified gaps. Our aims were to assess the disease framework and understanding of PTB and periodontal disease by professionals and community members. Our rationale for including questions pertaining to periodontal disease stems from a well-documented association between maternal oral health and risk of preterm birth and low birthweight infants. These findings have been replicated in both industrialized and rural and low resource settings [11-18]. Following our initial assessment using qualitative approaches, we aimed to partner with the Ministry of Health and regional obstetrical and oral health experts. The overarching goal of this mentored partnership was to develop tailored educational materials and readily deliverable health care messages for community health workers and clinicians to assist them in effectively educating pregnant women about PTB and oral health including defining terms, underlying etiologies, causative models, and risk associations; convey current knowledge about prevention of PTB and periodontal disease in a culturally appropriate fashion; and provide knowledge regarding anticipated management and outcomes specific to preterm delivery.

The main goal or purpose of this study was to qualitatively assess baseline knowledge and perceptions regarding preterm birth (PTB) and oral health in an at-risk, low resource setting surrounding Lilongwe, Malawi. The primary, secondary and tertiary aims were to determine what is understood as to the normal length of gestation and how gestational age is estimated, to identify common language regarding PTB, and to assess what is understood as to options for preterm labor management. As prior qualitative research had largely focused on patient or client-based focused groups, we also included groups largely comprised of community health workers (CHWs) and providers.

## Methods

### Sites and recruitment

**Figure 1:**
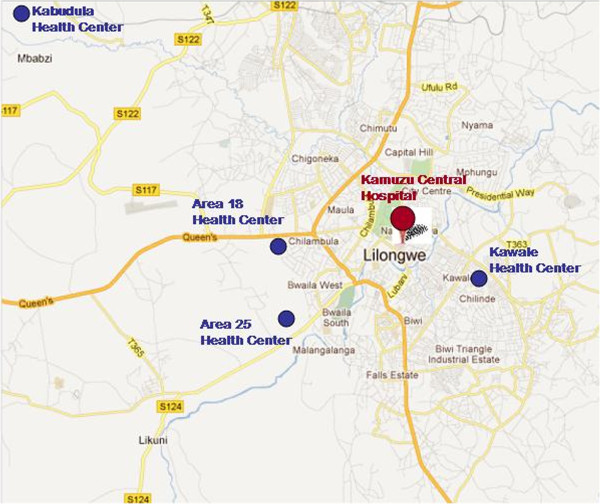
**Map of region where qualitative research sites were located.** Shown is the location of the central referral hospital, Kumuzu Central Hospital (KCH), Lilongwe, Malawi. Sites included Area 25 Health Center, and Kabudula Health Center. *Googlemaps.com.*

All studies were undertaken following IRB approval with the National Health Sciences Research Committee with the Malawi Ministry of Health (IRB00003905, FWA00005976). Baylor College of Medicine/Texas Children’s Hospital has a well-established pediatric HIV clinic in Lilongwe, Malawi on the campus of Kamuzu Central Hospital which is operated as part of the Baylor International Pediatrics Aids Initiative (BIPAI, Baylor-Malawi). The pediatric program has extensive outreach to multiple health centers on the periphery of the city (as far as 52 km from the center of town in the region of Kabudula; Figure 1). They support a community health worker network that provides prenatal and family planning education for women (both with and without HIV) while awaiting their antenatal visits. This program delivers health care messages which are in alignment with the Malawi Ministry of Health programs, and focuses on initiatives deemed of highest national importance. For women with HIV, the community health workers conduct home visits. Through the supervisory director of *Tingathe* (meaning “we can do it!” in the local language of Chichewa) and center head of the community health workers, a series of focus-group discussions and in-depth interviews with key informants at two large government health centers (Area 25 and Kabudula; Figure 1) were arranged weeks in advance. Area 25 and Kabudula are district health centers which are part of the referral network to the designated tertiary care obstetrical hospital (Kamuzu Central Hospital, Ethel Mutharika Maternity Ward); approximately 5100 deliveries occur annually at these sites. All potential subjects (including current gravidae) were informed that their participation was voluntary, and were provided with an overview of the discussion but not material nor questions per se. Volunteers were compensated for their travel expenses, but other than samples of xylitol mints, candies and gums were not remunerated for their participation. In accordance with the IRB-approved protocol, verbal informed consent was obtained prior to initiation of each focus group discussion, as well as with each in-depth interview. All participants were informed that the study was approved by the Health Sciences Research Committee with the Malawi Ministry of Health, and a copy of the approval letter was made available. The investigators explained that the overarching study is supported by USAID as part of a larger project aimed at Saving Lives at Birth.

### Study design

An investigative team comprised of trained female clinician investigators (n = 2), a data recorder, and a minimum of two interpreters (range 2–4, both male and female) held six focus groups for voluntary participants (n = 33) on consecutive days. Participants included CHWs, patient couples (gravid and recently post partum), nurse midwives and matrons, and clinical and medical officers at the two rural health centers (Table 1). The working definitions of focus groups and semi-structured interviews were based on the terminology as defined by Morgan [7]. Semi-structured questions facilitated discussion on pregnancy, preterm birth, and periodontal disease (Table 2). Prior to initiation of focus groups, all questions were vetted by the clinician investigators, including U.S. based investigators (J.L. and K.A.) and regional obstetrical specialists (equivalent to ABOG-trained and boarded obstetrician gynecologists, G.C. and H.P.). Baseline semi-structured questions included definitions, perception, causation, management, and accepted interventions (Table 2). We had been advised that having groups comprised of both men and women might lead to the men dominating the conversation; conversely, women may have chosen not to participate if their husbands could not also be present. Therefore, in addition to recording the content of their responses, we opted to track the number of responses from men and women; this data is presented as a footnote to Table 1.

**Table 1:** Voluntary subject participants and methods of data collection

**Method of data collection**	**Number of groups/interviews**	***n *subject volunteers**
***Focus group discussions (total time: 110 minutes at site 1, 85 minutes site 2)***		
**Mothers**^**#**^	2 groups (couple interviews)	4
**Fathers**	2 groups (couple interviews)	4
***Key informant interviews***		
**Community health workers**	2 group interviews	20
(total time: 120 minutes site 1, 115 minutes site 2)		
**Nurse midwife/matron**	3 group interviews	3
(total time: 40 minutes site 1, 65 minutes site 2)		
Clinical officer*	2 individual interviews	2
(total time: 60 minutes site 1, 65 minutes site 2)	1 group interview (with midwives, site 2)	

An independent study volunteer and the primary investigators (K.A. and J.L.) manually and digitally recorded both the time, number of responses, and *verbatim* nature of the responses. These tabulations were then internally checked, and concordance and consensus was reached on the same day as the interview occurred. Further clarification regarding language, statement, or contextual inference was achieved using an interpreters. This was sought either prior to close of the group interview, or by the end of the same day as the interview occurred.

^#^All women (mothers) were multiparous, and reported having experienced the anticipated spectrum of comorbidities for this global region. This might include HIV seropositive, history of pregnancy loss, preterm birth, and miscarriage, preeclampsia/eclampsia, and anemia from malaria. There were both currently gravid and non-pregnant volunteer subjects included. This information was not formally collected nor reported in order to protect subject confidentiality, but may have been shared during the course of the group discussions. At site 1, 87.6% of answers were given by women/mothers, and 13.3% were given by men/fathers. At site 2, 55.6% of answers were given by women, and 44.3% by men.

*Clinical officers are somewhat akin to a community practice physician or advanced physician assistant, typically with two years of formalized training followed by18 months to two years of multidisciplinary internship. Clinical officers are well-versed in prevalent complications in obstetrics and gynecology and common interventions, including cesarean deliveries and assisted deliveries, dilation and curettage, and basic ultrasound.

**Table 2:** Discussion group and key informant questions

**Topic**	**Discussion group and key informant questions**
***Definitions***	
**Gestation**	What is the normal length of pregnancy?
	How does a woman know when she is due?
**Preterm birth**	When is a baby born too soon?
	What if a woman has pains months before the due date?
**Viability/neonatal mortality**	What is the earliest age in pregnancy that a baby can survive?
	What is the lowest weight a baby can survive? What do most babies weigh if they are term? Preterm or born too soon?
***Epidemiology, Etiology and Risk Association***	
**Preterm birth prevalence**	How common is preterm birth in Malawi?
	Do you know women who have had a baby born too soon?
**Preterm birth etiology/explanatory models**	What are causes of preterm birth?
	What are causes of early labor pains? Vaginal bleeding?
**Oral health**	What is usual tooth and oral health care?
	How do most women receive oral health care? In pregnancy?
***Infrastructure and Access to Care***	
**Antenatal care**	When do most women come for antenatal care?
	Why do some women not come for antenatal care?
**Preterm birth care**	What can health facilities do if a woman presents with preterm contractions? Do women go to a health facility with preterm labor?
	Have you heard of medicines to make a baby’s lungs mature early (steroids) or ways to prevent PTB (progesterone or cerclage)?
	If available, how might women prefer to take a medication to prevent preterm birth (daily vaginal suppository versus weekly intramuscular injection)?
**Delivery site**	Where do women go first if they have preterm labor or bleeding?
	Where are women referred if they have preterm labor or need treatment because a baby is born too soon?
***Openness and Acceptability of New Concepts or Potential Preventions/Interventions***	
**New concepts**	What if I told you that there was an association between preterm birth and oral health/gum/periodontal disease? Would you believe me?
**New approaches**	Would women in Malawi chew gum? Eat mints or candies?

In order to enable iterative data analysis, questions were followed up on in logical formats as new issues or evident need for clarification arose. Male and female interpreters fluent in Chichewa and English were utilized for both translation/interpretation during the discussions and interviews, and later with iterative analysis.

### Primary goal

Our primary goal was to examine core knowledge and constructs (formal and informal) of preterm birth and prevention among patients/clients and their partners/husbands, CHWs, and key informant providers (midwives and clinical and medical officers). We sought to understand 1) perceptions of normal length of gestation and how gestational age is estimated, 2) common language regarding PTB, and 3) perceived access to frameworks surrounding management of PTB.. These served as our primary, secondary, and tertiary aims of the study, respectively.

### Data collection, management, and analysis

An interview guide was developed with lead-in questions listed in Table 2. The names, ages, and any potentially identifying information of volunteers were not recorded to protect anonymity. Questions were asked in English, and interpreted/translated by native-speaking and knowledgeable personal (bilingual CHWs and nurses/matrons) into Chichewa, the local language. Responses were given in both English and Chichewa, and all answers were interpreted/translated and recorded. Both investigators performing the interviews recorded the responses in writing and compared their written reports at the end of each group discussion. As previously described [19], to improve trustworthiness in the qualitative data and interpretations, source data was triangulated both between and among focus groups and key informant interviews. Iterative data analysis was carried out employing a framework analytic approach [20-24]. A framework for data analysis was developed using lead in questions and follow up questions. Systematic application identified major and minor themes, which were followed from the first focus group forward.

### Application of findings

All members of the research team debriefed both verbally and in written form in order to reflect on and validate findings. Key phrases and illustrative comments were compared and reviewed among research team members, and additional interpretation/translation was utilized as necessary. Dissemination discussions were undertaken with regional physicians and clinical officers, and later with health care workers hired through the primary grant funding. Information and knowledge gaps were shared through daily health care worker messages, one-on-one care, and with the broader community of providers through antenatal clinic speeches, drama, song, and in a subsequent provider-only “Open Day” continuing education workshop.

## Results

Table 1 provides an overview of the discussion group participants and key informants, and Table 2 presents the initiating standardized questions. We observed that women provided the majority of answers to questions, notably when the questions occurred as discussion groups with couples (site 1 87.6% provided by females; site 2 55.6% by females). This was true for both directed and non-directed questions. For example, at site 1 eight questions were directed to fathers in the discussion group but five of these eight questions were also answered by the mothers. This increased our confidence in hosting discussion groups as couples, and alleviated the need for expanded single sex discussion groups. Total time in direct volunteer subject interaction was 660 min; allotment breakdown by group and key informant is provided in Table 1. The order of interview group and key informants was varied by site and day of interview.

Table 3 summarizes the responses of the patient couples, CHWs, and the clinical and medical officers and midwives and matrons in an interpretive framework. In total, 162 directed and non-directed questions were asked and 298 answers were given. The ratio of answers to questions did not change appreciably between the sites (1.59:1, 2.03:1). The emerged major and minor themes similarly did not vary between sites, despite an over 50 km distance separation, lack of overlap by site and integer time of training of key informants, and baseline variation in key incidents among couple participants.

**Table 3:** Iterative responses by discussion group or key informant, highlighting emerged major and minor themes

		**Patient couples**	**Community health workers**	**Clinical officers/midwives**
**Definitions**	**Length of a normal pregnancy**	–9-10 months	–36 weeks, which was considered the equivalent of nine months	–40 weeks
	**How due date is known**	–Just know the month/not the date	–By last period and first antenatal visit	–LMP with gestational wheel
		–The midwife tells you from a wheel	–Most know the month but not the week of LMP	–Ultrasound (“best done at 28 weeks so you can tell due date and presentation of baby”)
		–Only by coming for antenatal care and being seen by a clinician who palpates abdomen	–Not aware of ultrasound for dating a pregnancy	
	**Earliest age a baby can survive**	–6 months if delivered at a health facility (higher chance of baby dying if delivered at home)	–6 months maybe, 7 months yes	–7 months
			–28 weeks (which was estimated to be 2.3 kg)	
			–1.5-1.9 kg	
		–1 kg	–1 kg if in an incubator followed by Kangaroo Care	
		–2 kg		
**Explanatory models**	**Causes of preterm delivery**	–Beaten by husband	–Stress	–Twins
		–STIs (AIDS, syphilis, gonorrhea, hepatitis)	–Hard work	–Anemia
		–Malnourishment	–Being beaten	–Close spacing of pregnancies
		–Placenta problems	–Malaria	–Overwork
		–Maternal sickness	–Other sickness	–Infections like malaria and STIs
		–Mother too young (<18) or too old (>45)	–Having had many children	–Trauma
			–History of abortions	–Young age
				–Multiparity
				–Incompetent cervix
**Access to care**	**Actions to take if preterm contractions**	–Go to health center	–Go to health center (though might not tell family they are going if it is not a scheduled visit)	–Refer to district hospital
		–Go to hospital—which could be 12–20 km away (walk or bike or use ox cart or tractor from nearby estate)	–Might call CHW (20-25% of families have phones)	–Refer to central hospital
	**Reasons not to go to health center or hospital**	–Church might not condone going to a health center	–Fear of “being cut with sharp things”	–Women and their families may not be able to arrange or afford transport
		–Traditional healer might have herbs to stop contractions	–Fear that nurses and clinicians will be harsh	–“Nothing can be done”
			–Transportation	
	**Services available at health facilities to treat preterm labor or prevent recurrent preterm birth**	–Kangaroo Care	–Rest	–Referral to district hospital
		–Medications to stop contractions	–Cesarean to save mother and baby	–Clinical officer: Not aware of availability of steroids to accelerate fetal lung maturity
		–Never heard of steroids or progesterone	–Referral to district hospital	–Clinical officer: Give salbutemol (terbutaline) and refer if contractions do not subside
			–Never heard of steroids or progesterone	–Referral hospitals offer Kangaroo Care, a nursery, and dexamethasone for mother in preterm labor and cerclage for woman
				–Never heard of progesterone
**Openness and acceptability**	**If progesterone available, route of delivery preference**	–Vaginal route (cost of transport to hospital for weekly injection is too high)	–Vaginal route	–Injection (can document adherence to regimen)
	**Openness to new concepts**	–Would believe that gum disease can cause PTB if given an explanation and the doctor said it was true	–Would believe gum disease can cause PTB “but, first, you have to tell us why”	–“I do not know that gum disease may cause PTB, but if you explain it, maybe I would believe it.”
			–“We would need ‘community sensitization’ first before women will believe this.”	
			–Want to make sure previous studies have been done, no harm is being done, and “Malawians are not being used as guinea pigs”	
	**Social acceptability of new approaches**	–Gum or mints are ok	–Gum or mints are ok	–Gum or mints are ok
		–Prefer a jar of gum over a package (can use later for storing other things like salt or medicines)	–Packages are easier to keep in a safe place but jars could be used later for storing sugar	–Have heard of some chewing gum reducing tooth decay
	**Optimal delivery sites if preterm labor**	–District or central hospital	–District hospital unless free transport is available to central hospital	–District hospital (they will decided if to refer on to central hospital)

To improve trustworthiness, all data was compared and findings were triangulated. All members of the research team including interpreters “debriefed” after each interview or discussion group to enhance the confirm ability of the findings through the representative ranges of perspectives, personalities, and a priori knowledge of the team members.

The ratio of questions asked to answers given were tracked by data recorders. At site 1, 71 questions were asked in total, and 113 answers were given at a ratio of answers to questions of 1.59:1. At site 2, 91 questions were asked and 185 answers given (2.03:1).

### Normal length of gestation and how gestational age is estimated

Normal length of gestation was most commonly described by men and women from the community as 9–10 months, by CHWs as 36 weeks, and by clinicians as 40 weeks, which reflected the same discrepancy in definition we see in the United States: most people interpret a month as equivalent to four weeks, which leads many to conclude that 40 weeks gestation is ten months, or that nine months is 36 weeks. Patient couples and clinicians referred to last menstrual period and use of a gestational wheel to determine the estimated due date (“the midwife tells you from a wheel”); CHWs and patient couples pointed to measurements at the first antenatal visit as determining the due date (“by coming for antenatal care and being seen by a clinician who palpates the abdomen”). Clinicians mentioned ultrasound though most CHWs were not aware of the role of ultrasound for dating a pregnancy. Of interest, clinicians stated that 28 weeks was the best time to do a dating ultrasound (in the United States we prefer the more accurate dating of early pregnancy) but they explained that the advantage of a 28 week scan was that it also provided documentation of the fetal presentation (obstetricians might argue that presentation is best assessed within one month of due date).

### Common language regarding preterm birth

Definitions of preterm birth or “born too soon” varied widely among interviewees: “six months” and “less than one kilogram” were considered at low chance for survival by most participants. Normal estimated gestational weight at 28 weeks or six months ranged from one kilogram to 2.3 kilograms. Causes of PTB were consistent among the three groups (couples, community health workers, and clinicians) and included domestic violence, overwork, maternal illness, sexually transmitted diseases, and young age. Clinicians tended to also include multiple pregnancies and short interval between pregnancies as possible etiologies.

### Understanding of options for management of preterm birth

All participants agreed that going to the health center or district hospital was an appropriate action to take if preterm contractions occurred. Although there had been a recent regional recommendation to send all women with preterm contractions to the central hospital, most participants including clinicians were unaware of the new policy. Common barriers to leaving home included transportation, fear of the hospital and disrespectful treatment by hospital staff, and belief that nothing could be done if preterm labor occurred. Members of couples did mention that perhaps traditional healers would have medication to stop contractions. Patient couples and CHWs generally had not heard of the advantage and the availability of antenatal steroids to accelerate lung maturity; one clinical officer was equally unaware of this option. The concept of intramuscular or intravaginal progesterone to prevent PTB in a woman with a history of prior PTB as well as the possible role of good dentition in preventing PTB were new to all participants. However, the most common response to hearing this and other new information was: “Explain to us why and how this works and we might believe you”.

## Discussion

Qualitative assessment of perceptions surrounding preterm birth in Malawi, the country with the highest preterm birth rate in the world [1-5], has been limited. Tolhurst et al. [19] found that women (recruited from both health centers and from villages) differentiated miscarriages as occurring up to six months gestation and preterm birth at seven to eight months; common causes were believed to be “modern” illnesses such as sexually transmitted infections (syphilis, gonorrhea, and AIDS), malaria, and anemia; traditional illnesses (*libale*, *mauka*, and *likango*) associated with growths or sores on the genital area; witchcraft; impurity related to sexual practices and who has cooked a pregnant woman’s meal; heavy work; and “bitter medicines” (especially traditional medicines since they are not measured). Our interviews elicited similar definitions of preterm birth and several similar causes with the exception of witchcraft and traditional illnesses.

We report the perceptions of parents and gravidae, as well as the views of community health workers, nurse midwives, and clinical officers. Our findings can be summarized by four major themes which emerge: [1] *community conceptualization and definition* of term and preterm birth, including dating of pregnancy; [2] *explanatory models and etiology of preterm birth* iterating association and causation in a disease framework; [3] *perceived access and barriers to care and prevention/interventions* with identified gaps in knowledge or communication between stakeholders*;*[4] *willingness to consider novel preventions/interventions and alternative explanations.*

### Community conceptualization and definition

Tolhurst et al. have described their linguistic based interpretations and translations pertaining to how participants distinguished miscarriage (as they had interpreted in Chichewa, *chitayo*) from preterm birth or born too soon (in Chichewa, *kuchila masika asankwane*) [19] This latter definition was defined by these authors as indicative of the preterm birth where the infant may be born alive and is fully formed, and is further distinguished from an infant or baby (*khanda,* or *mwana wakhanda*). Our interpreters, translators, clinical officers, midwives and matrons, and community health workers translated these terms from the rural regions surrounding Lilongwe, Malawi slightly different. Collectively, their interpretation of English to Chichewa for abortion or miscarriage prior to 28 weeks was translated as *kupita pachabe* or *mtayo*, while fresh stillbirth is *mwana wakufa* and macerated stillbirth is *mwana wakufa osupuka*. In our region of Malawi under study, *chitayo* refers to when a man has intercourse with a woman who has just had a miscarriage. Regardless of the colloquial distinctions, the fact that there are unique, distinct, and specific terms for each of these pregnancy outcomes is significant. While largely descriptive, each of these terms are clinically predictive in nature and therefore indicative of community held distinctions with long-standing origin and discrete predictive and explanatory variables [20,21].

All respondents were aware that a full term pregnancy lasted 9 completed months, or through to the 10^th^ month, but conceptualization in weeks of gestation was more common to midwives and clinical officers; community health workers perceived that term gestation was at 36 weeks or the equivalent of the start of the 9^th^ month. The majority of couples perceived that the due date was known by the month and one of the purposes of attending antenatal care was to determine the month the baby was due (“the midwife tells you from the wheel”, the “clinician palpates the abdomen”). It was the perception of interviewed community health workers that most clients or patients knew the month but not week of the last menstrual period (LMP) and few women kept calendars. All providers were aware of the utility of ultrasound, and at one of the two sites ultrasound was performed on every antenatal care client “best done at 28 weeks so you can tell due date and presentation of baby”. One clinical officer was aware that the earliest ultrasound was best for distinguishing term and preterm birth.

Definitions of viability were broad and gaps in knowledge among all participants were identified. Specifically, couples proved a range of 6 months of survival (“if delivered at a health facility, with a higher chance of the baby dying if delivered at home”), with all couples giving a discordant range of weight (1 to 2 kg) to age (6 months, or 24 weeks). This discordance persisted among key informants. Community health workers stated “6 months, maybe; 7 months, yes” and “28 weeks, estimated to be 2.3 kg”. Additional weight ranges were 1.5 to 1.9 kg, or “1 kg if in an incubator followed by kangaroo care”.

### Explanatory models and etiology of preterm birth

We were unable to corroborate the findings of Tolhurst et al. [19] regarding witchcraft causing a pregnancy to disappear. Similarly, “impurity” from sex, death, blood and cooking was not perceived nor mentioned as a cause of preterm birth. In contrast, at both sites (including a distal rural site 50 km north of Lilongwe, Kabudula Health Center), illness categories and etiology structures were viewed as commons causes and risks for preterm birth. All participants and key informants identified preventable illness and sexually transmitted infections (HIV/AIDS, syphilis, gonorrhea, hepatitis, malaria, ‘other sickness’) as potentially most likely causative. Preventable or modifiable risks included “being beaten by husband”, hard work/stress, overwork, trauma, and “mother too young or too old”. Malnourishment and anemia were identified among causative maternal comorbidities, as well as close spacing of pregnancies, having had either too many children or too many abortions or miscarriages, and malnourishment. Clinical officers and midwives perceived twins, grand multiparity, and incompetent cervix as prevalent risk factors, and commented on the importance of contraception and spacing for prevention and management of preterm birth. All participants knew of “many women” who had experienced a preterm birth, distinguished the preterm birth from a miscarriage, and estimated the rate overall in the population to range from “1 in every 4 mothers, or maybe 1 in every 5”; few participants could convert this to a percentage. All clinical officers and midwives knew of women with recurrent preterm birth, and distinguished from recurrent pregnancy loss or miscarriage.

Taken together, these framework explanatory models are potentially crucial to acceptance of preventative and intervention strategies, and suggest a largely biomedical construct and definition for preterm birth. This would further suggest that implementation of preventative measures and interventions currently employed in Western medicine may be predicted to be successful if logistical barriers can be overcome.

### Perceived access and barriers to care and prevention/interventions

We observed that there was discrepant understanding between providers and patients regarding perceived access and barriers to care. For example, the usual pattern of referral in Malawi is from local health center, to district hospital, and, if needed, from the district hospital to the central hospital. However, a national recommendation from the Malawi Ministry of Health issued one year prior to our investigation mandated referral of all women in possible preterm labor directly to Kamuzu Central Hospital, the tertiary care hospital, in order to avoid time delays of multiple transfers. In the course of our qualitative research we discovered that the health center clinicians, midwives, and community health workers were unaware of the recommendation. Ergo, all participants perceived that the “proper referral pattern” for preterm labor patients was first to the district hospital (which may have been less likely to have a steady supply of antenatal steroids and did not have a steady presence of a qualified pediatrician or advanced care nursery). Details regarding perceived actions to be taken, reasons to go or not got to a health center or hospital, and available services and perceived service gaps are detailed in Table 3. We as the authors of this study are not attempting to make recommendations to the Ministry of Health regarding whether proven efficacious interventions (such as antenatal steroids or progesterone for the prevention of recurrent preterm birth) should (or should not) be available at local health centers or district hospitals; however, the availability and proven utility should be clearly communicated to all levels of providers and patients. As a second example of the lack of concordance, one incidental finding in our study was the perception that “zero” women (perceived as true by clinical officers) used traditional birth attendants (TBA), versus “half of women” (perceived as true by community health workers and couples) frequented births with TBA.

### Willingness to consider novel preventions/interventions and alternative explanations

All participants were willing to consider innovative or novel measures, as well as alternative disease model explanations. For example, when both couples and community workers were informed “what if I told you that periodontal or gum disease is associated with a higher chance or risk of preterm birth, would you believe me?” the majority response was “yes, if you told me why”. Similarly, when given stated benefit of interventions such as progesterone for prevention of recurrent preterm birth, all participants stated they would be willing to prescribe, refer, or use. Of interest, both vaginal suppositories and i.m. injectable forms of progesterone were deemed acceptable or perceived to be acceptable. However, key informants thought it “better” for compliance if i.m. to assure use, while couples and community health workers perceived higher likelihood of compliance with home vaginal suppositories (“most women cannot afford the transportation’).

Strengths of our study include both the inclusion of patients, CHWs, clinical officers, and midwives at their site of care and in their native language, as well as the use of focus groups and key informant interviews to gather data as well as to educate and empower those who are the subjects of the research [22-25]. In addition, this is the first study to expand the discussion of perceptions surrounding preterm birth in Malawi to include an understanding of what interventions might be available at health facilities. As measured by a nearly 2:1 ratio of responses to questions, the discussion was robust and bilingual interpreters enabled both triangulation of responses as well as translation. Limitations to our study include a relatively small participant sample size at 33. However, we had many overlapping responses, suggesting that we were close to saturation (the end point of many qualitative studies, when questioning of additional subjects yields no new responses; 9,10, 19–24). We were initially concerned that mixing men and women in the focus groups might limit women’s responses; however, in addition to the women responding to questions more frequently than the men, the degree of spontaneity with which the women answered gave us the impression that the women were not censoring their words. In addition, Caucasian U.S. physicians facilitated the focus groups with patients, which might have discouraged discussion of traditional beliefs. This was likely mitigated by the fact that both physicians were obstetricians/maternal-fetal medicine specialists who had significant experience as global health providers and had worked in the region intermittently for 1 year. Focus groups, including ours, have inherent limitations which include the risk of interviewers giving out unconscious cues to suggest certain answers and the data may be challenging to analyze. An additional potential limitation included the fact that all of our participants were recruited through their health center, which may have preselected for a more educated group of subjects than those in the Tolhurst study (where individuals were recruited from both health centers and villages [19].

## Conclusions

All participants were aware of many women who had babies which were born too soon, and felt that this was a significant health concern which needed to be addressed. We were able to identify gaps in knowledge among community members, CHWs and clinicians regarding normal length of gestation, definitions of PTB, and options for management of preterm contractions or a prior history of PTB. As a result we.are now engaged in invited partnership and establishing ongoing venues for education, clinical care, and outcomes-based research to be translated into meaningful interventions. This partnership includes both out of country and in country expertise, and with ongoing input from antenatal care community health workers, midwives, clinical officers and midwives we have made significant strides in addressing the identified gaps including the following:

### Open day

Given our finding of knowledge gaps pertaining to preferred referrals patterns in this low-resource setting, a one day workshop was held for 50 health workers from 4 health centers and 2 mid-referral district hospitals. The goals of this Open Day was to begin to close these knowledge gaps, and provide education by Kamuzu Central Hospital physicians (Malawian obstetricians and dental surgeons) about preterm labor, oral health, and the possible link of periodontal health to duration of pregnancy. Of note, we surveyed Open Day participants at the start of the activity and after its completion. Despite the Malawi Ministry of Health mandate one year prior, while only 52.5% would have recommended Kamuzu Central Hospital (KCH) to family or friends prior to the Open Day event, 93% would after the event.

### Patterns of referral recommended by the district health officer have been clarified and are being utilized

In the past and prior to Open Day, health centers referred to the district hospital, which then decided which patients need transfer to the central (tertiary) hospital. Now, any woman with possible preterm labor will be sent directly to the central hospital (KCH), which has the most reliable supply of maternal steroids for acceleration of pulmonary maturity and prevention of preterm morbidity and mortality. This has further focused procurement, and enabled consistent use by accepted WHO gestational age norms [23].

### Addressing identified knowledge gaps

Teaching materials for community health workers were developed in collaboration with regional specialists and programs through the Malawi Ministry of Health to include information on interventions available for women with a history of previous preterm deliveries (vaginal progesterone) and for women in preterm labor (antenatal steroids). Now, our CHWs adapt the messages for oral presentations, in song and in drama, to women awaiting antenatal clinic visits. They specifically address the gaps in knowledge identified in our qualitative research. Examples include: “**What is preterm labour?** Preterm (or premature) labour (onset of labour before 37 weeks since the last period) is a common but serious complication in pregnancy and may be associated with death of the infant close to delivery. The preterm birth rate in Malawi is high; one out of every five babies is born one or more months prior to the expected delivery date”, and “**What can every woman do to prevent preterm labour?** Get early treatment for illnesses such as malaria, HIV, and STIs. See a doctor for treatment of high blood pressure, diabetes and other chronic conditions before you get pregnant. Eat vegetables, fruits, grains, and meat or nuts daily. Clean teeth twice a day with toothbrush. See a dentist if you are having dental problems. Get help if your husband is beating you. Go to a health center to get a family planning method so you do not get pregnant soon after a birth”. Although we realize that access to all of these recommendations may be limited within the context of a low resource setting, we worked with CHWs who felt that these messages were appropriate; for example, they explained to women how they could add commonly available nuts to their meals to increase protein intake (even if meat was not available). Other messages involved conveying “**What can be done if a woman has premature contractions?** Medication (dexamethasone or betamethasone, known as steroids) is available at hospitals to improve the baby’s chance of survival even if born too soon. Deaths of preterm infants can be prevented without intensive care but with measures such as antenatal steroids, neonatal resuscitation (“Helping Babies Breathe”) and Kangaroo Care. **Where is there specialized care for women who have had a premature birth or premature labour?** Clinicians at a specialist antenatal clinic can evaluate women with a prior PTB for possible treatment with vaginal progesterone (daily vaginal tablets for 3–4 months) or a suture to the cervix. Women in premature labour may be admitted to the hospital for injections of dexamethasone (four in 48 hours) or betamethasone (two in 24 hours) to accelerate the readiness of the baby’s lungs to breathe if he or she is born early. Please talk with your provider today to help you with this referral if you are concerned”.

### Every birth counts

Given that we had observed discrepancies between perceived use of community health centers and rate of preterm birth, we have worked with our CHWs to make “every birth count”. Ongoing in-person data acquisition at an expanded cohort of community health centers and surrounding villages will enable improved statistics as to the rate of preterm births, term births, fresh stillbirths (neonatal demise), and macerated stillbirths [22-25].

In summary, qualitative research into community baseline gaps and needs is beneficial. Applied and informed dynamic education and knowledge dissemination, accepted prevention-intervention strategies, and inpatient and outpatient referral networks are under development as a result of this study. Further investigation of the role of traditional birth attendants and the possible need to engage broader community leaders and key informants in preterm birth prevention efforts is anticipated to be of critical importance moving forward.

## Abbreviations

PTB: Preterm birth; CHW: Community health worker; IRB: Institutional review board; BIPAI: Baylor pediatrics aids initiative; ABOG: American board of obstetrics and gynecology; TBA: Traditional birth attendant.

## Competing interests

The authors declare that they have no competing interests. Findings were presented in a poster at the 33^rd^ Annual Meeting of the Society for Maternal Fetal Medicine, San Francisco, California, February 11–16, 2013.

## Authors’ contributions

KA conceived of the overarching study. JL and KA designed the qualitative research study, carried out the research, analyzed the data, and wrote the manuscript. DN coordinated all aspects of the research, and DN, GC, PN, and HP provided interpretive services. GC, PN, HP and SR assisted in study design and implementation planning and edited the manuscript. All authors read and approved the final manuscript.

## Authors’ information

JL, KA and SR are U.S.-based ABOG boarded obstetrician/gynecologists and maternal-fetal medicine specialists (KA, SR) with global health experience in Malawi and other under-resourced regions. GC and HP are Malawian obstetrician-gynecologist specialists, and GC is the Department Head at Kumuzu Central Hospital; at present, there are fewer than 20 Malawian obstetrician gynecologist specialists in country. PN is a Malawian pediatrician, and DN is a Malawian community health worker. In addition to their medical doctorate degrees, JL has been conferred an M.P.H. and KA a Ph.D. and M.S.C.I.

## Pre-publication history

The pre-publication history for this paper can be accessed here:

http://www.biomedcentral.com/1471-2393/14/123/prepub
